# Spatial Principles of Chromatin Architecture Associated With Organ-Specific Gene Regulation

**DOI:** 10.3389/fcvm.2018.00186

**Published:** 2019-01-15

**Authors:** Douglas J. Chapski, Manuel Rosa-Garrido, Nan Hua, Frank Alber, Thomas M. Vondriska

**Affiliations:** ^1^Departments of Anesthesiology, Physiology and Medicine, David Geffen School of Medicine, University of California, Los Angeles, Los Angeles, CA, United States; ^2^Molecular and Computational Biology, Department of Biological Sciences, University of Southern California, Los Angeles, CA, United States

**Keywords:** transcription, chromatin conformation capture, genomics, chromatin structure, epigenetics

## Abstract

Packaging of the genome in the nucleus is a non-random process that is thought to directly contribute to cell type-specific transcriptomes, although this hypothesis remains untested. Epigenome architecture, as assayed by chromatin conformation capture techniques, such as Hi-C, has recently been described in the mammalian cardiac myocyte and found to be remodeled in the setting of heart failure. In the present study, we sought to determine whether the structural features of the epigenome are conserved between different cell types by investigating Hi-C and RNA-seq data from heart and liver. Investigation of genes with enriched expression in heart or liver revealed nuanced interaction paradigms between organs: first, the log_2_ ratios of heart:liver (or liver:heart) intrachromosomal interactions are higher in organ-specific gene sets (*p* = 0.009), suggesting that organ-specific genes have specialized chromatin structural features. Despite similar number of total interactions between cell types, intrachromosomal interaction profiles in heart but not liver demonstrate that genes forming promoter-to-transcription-end-site loops in the cardiac nucleus tend to be involved in cardiac-related pathways. The same analysis revealed an analogous organ-specific interaction profile for liver-specific loop genes. Investigation of A/B compartmentalization (marker of chromatin accessibility) revealed that in the heart, 66.7% of cardiac-specific genes are in compartment A, while 66.1% of liver-specific genes are found in compartment B, suggesting that there exists a cardiac chromatin topology that allows for expression of cardiac genes. Analyses of interchromosomal interactions revealed a relationship between interchromosomal interaction count and organ-specific gene localization (*p* = 2.2 × 10^−16^) and that, for both organs, regions of active or inactive chromatin tend to segregate in 3D space (i.e., active with active, inactive with inactive). 3D models of topologically associating domains (TADs) suggest that TADs tend to interact with regions of similar compartmentalization across chromosomes, revealing *trans* structural interactions contributing to genomic compartmentalization at distinct structural scales. These models reveal discordant nuclear compaction strategies, with heart packaging compartment A genes preferentially toward the center of the nucleus and liver exhibiting preferential arrangement toward the periphery. Taken together, our data suggest that intra- and interchromosomal chromatin architecture plays a role in orchestrating tissue-specific gene expression.

## Introduction

Before DNA was universally recognized as the genetic material, it was hypothesized that nuclear proteins may be responsible for how the same DNA does different things in the various cell types of a multicellular organism (1). Since around the same time, it has been appreciated that nuclear proteins, histones in particular, exhibit distinct biochemical properties across cell types and stages of development (2)—DNA itself has long been known to be modified by methylation according to similar physiological variables (3). In the ensuing decades, it has become clear that histone modification and nucleosome positioning play a central role in specifying distinct transcriptomes (4, 5), but the implications for chromatin structure have remained uncertain.

More recently, the emergence of chromatin capture technology combined with next generation sequencing has enabled unprecedented analyses of endogenous chromatin structure with increasing levels of resolution (6–8). Chromatin compartmentalization has been characterized as an intrinsic property of nuclear architecture, denoting regions tending to be more accessible as “compartment A” and those less accessible “compartment B” (7). In addition to compartmentalization, Hi-C data can reveal properties of chromatin looping (9, 10). Putative gene loops have also been identified from RNA Polymerase II ChIP-seq datasets (11), wherein genes have their promoters and transcription end sites in close 3D proximity to facilitate continued transcription. Folding of the genome is a non-random, reproducible process that favors local over long range interactions. This behavior leads to the formation of topologically associating domains (TADs), which exhibit greater interactions within themselves than between, constituting a structural unit greater in scale than the nucleosome (TADs are composed of kilobases of DNA and associated nucleosomes) and smaller than the chromosome, with boundary regions between TADs being ostensibly responsible for cordoning distinct regions of transcriptional behavior. HiC, one of the principle techniques for genome wide chromatin structural analysis, has now been deployed in multiple laboratories around the world, as well as in multiple cell types, revealing TADs and chromatin compartmentalization to be conserved structural rules governing genome organization (12, 13).

These observations raise the following question: if TADs are a conserved feature of epigenomes across cell types, where does the specificity in structure arise? Compounding this question is the fact that, until recently, chromatin conformation capture studies have been often carried out in either cell culture or whole tissue extracts, making it possible to evaluate neither terminally differentiated cells nor the cell type-specific nature of chromatin structure. While we understand transcriptome changes across multiple organs and disease states, a major gap in our basic understanding of organ function is how genome architecture varies between cells and how this relates to gene expression.

To address these gaps in knowledge, we investigated chromatin structural differences between heart and liver, and how they relate to tissue-specific gene expression programs. Specifically, we studied the role of genomic interactions (both intra- and interchromosomal) in organ-specific gene architecture. The analysis reveals a concordance between interaction frequency and organ-specific gene expression between tissues. We also explored compartment differences between organs, demonstrating that gene expression paradigms in distinct tissues act concertedly with their organ-specific compartmentalization pattern to regulate function of the cell. Lastly, we show that more interchromosomal interactions exist at organ-specific genes, and that about half of such interactions bridge distinct compartments within both cardiac and liver nuclei. Together, these investigations reveal organ-specific chromatin conformations that may contribute to cell identity in heart and liver.

## Materials and Methods

### Hi-C Bioinformatics

Hi-C datasets from this study were downloaded from NCBI GEO: Isolated cardiac myocyte data (14) were downloaded using accession number GSE96693 (Control_HiC). Liver data (acquired from isolated hepatocyte nuclei) (15) were downloaded using accession number GSE104129 (Hi-C reps1-5). This dataset comes from wild-type C57BL/6J mice whose hepatocyte nuclei were isolated via homogenization and then crosslinked in 1% formaldehyde in PBS and quenched in glycine (125 mM final concentration) for Hi-C. Hi-C libraries for both datasets were generated using Hi-C protocols based on (9) with small changes described in previous work (14, 15). Libraries for both heart and liver were constructed using MboI as the restriction endonuclease and sequenced deeply enough to achieve 5 kb resolution contact matrices (see Supplementary Table 1 for sequencing depth and valid interaction pair numbers, as determined by our pipeline described below).

Hi-C datasets were run through the HiC-Pro analysis pipeline (16), version 2.10.0. Briefly, raw FASTQ files from all biological replicates were combined for each organ, and aligned to mm10 using an MboI-digested restriction fragment list generated by HiC-Pro. After the quality_checks step, we built 5 kb resolution contact maps and performed iterative correction and eigenvector decomposition [ICE normalization, first described in (17)], using HiC-Pro. We also built 100 kb contact maps for 3D model building. We then converted contact matrices to a Fit-Hi-C (10) friendly format using the hicpro2fithic.py Python script provided with HiC-Pro, with the raw contact matrices and ICE biases as inputs. Fit-Hi-C version 2.0.3 was used to determine significant intrachromosomal interactions, using the following parameters: –b 200 –r 5000 –p 2. The advantage of Fit-Hi-C version 2 (as compared to version 1) is that it can determine significant intrachromosomal interactions without constraining the data to mid-range distances. That is, we can use Fit-Hi-C version 2 to identify regions of significant intrachromosomal interaction along *entire* chromosomes, at 5 kb resolution. Another benefit of Fit-Hi-C is that it reports a q-value for each interaction, and we can filter for significant (*q* < 0.01) ones. For significant interchromosomal interaction identification, we performed a similar Fit-Hi-C analysis, but with the following parameters to investigate interactions that are not on the same chromosome: –b 200 –r 5000 –x interOnly. Significant intra- and interchromosomal interactions (*q* < 0.01) at 5 kb resolution are quantified in Supplementary Table 1. A/B compartmentalization was calculated on 5 kb resolution contact matrices (14). For each bin in the genome, differences in A/B compartmentalization between heart and liver were noted and shown in Figure 3C. All analyses in this study were done on autosomes only, unless otherwise stated.

To generate 3D models of topologically associating domains, we first ran TopDom (18) version 0.0.2 on ICE-normalized 100 kb matrices to generate a list of TADs in cardiac and liver Hi-C data, using *window.size* = 3 as a parameter. We then used Population-based Genome Structure (PGS) software (19) to generate 10,000 3D models of the genome (autosomes + chrX), using default parameters, the 100 kb matrices, and TAD calls as inputs. The contact probabilities between TADs in the resulting population of genome structures are statistically consistent with the contact probability matrix from Hi-C experiments (Supplementary Figure 2). This resulted in an extracted list of *xyz* coordinates for each TAD that were used to generate PDB files for visualization, structure analysis, as well as distance matrix calculations to determine the closest interchromosomal TADs for each TAD in the genome (custom R scripts). For both heart and liver data, 100 kb resolution TADs were designated as being in compartment A or B based on the majority compartmentalization status of the 5 kb bins (A/B analysis described in paragraph above) that lie within each 100 kb resolution TAD.

### RNA-seq Bioinformatics

RNA-seq data from this study were downloaded from NCBI GEO: Isolated cardiac myocyte data corresponding to our previous study (14) were downloaded using accession number GSE96693 (Control_RNAseq Replicates 1–3). Mouse (C57BL/6) whole liver RNA-seq data from ENCODE Portal were downloaded using ENCODE Data Coordination Center accession number ENCSR000BYS (which is identical to the data at NCBI GEO accession number GSE90180). Raw FASTQ files from two biological replicates of liver tissue were downloaded. For both cardiac and liver RNA-seq library prep, rRNA was depleted and polyA selection performed. For the bioinformatics analysis, raw paired-end FASTQ files were aligned to the mm10 reference genome (Ensembl release 81) using HISAT2 (20) version 2.1.0 with an mm10 HISAT2 index (built in-house). Resulting SAM alignments were converted to BAM format and sorted by name with Samtools (21) version 1.7. Gene counts were determined using htseq-count (22) version 0.9.1, with the sorted BAM alignments and a GTF of known Ensembl genes from release 81 as input. The Bioconductor package DESeq2 (23) was then used to pre-filter the genes that have at least 10 reads between any of the replicates (3 heart and 2 liver) and to collapse replicates by organ with the collapseReplicates() function. Then, the counts() function with the normalized = T option resulted in a single normalized count value for all genes for each organ, for use during downstream analyses.

### Promoter-TES Analysis

Genes with promoter-TES loops were identified by determining, using the Bioconductor package InteractionSet (24), the genes that have significant (*q* < 0.01) Fit-Hi-C interactions with the promoter (−2,000 to +200 bp form TSS) *and* the TES of a gene. Genes that have such interactions underwent KEGG analysis using KEGG.db (25), a package in Bioconductor, with custom graphics generation using ggplot2 in R. Indicated *p*-values are calculated using a hypergeometric test.

### Organ-Specific Gene Designation

We designated organ-specific genes using the Human Protein Atlas (26), specifically the subset of genes that are enriched in heart and liver at the mRNA level. The Human Protein Atlas defines “tissue enriched” genes as having at least 5-fold higher mRNA expression in the organ of interest when contrasting against all other organs (26). Human Ensembl gene identifiers from these tables were fed into biomaRt (27) in R and converted to Mouse Ensembl identifiers. For further analysis, we filtered to keep gene coordinates on murine autosomes.

## Results

### Chromatin Microenvironments Facilitate Organ-Specific Gene Interaction

To examine whether nuclei of different cells create chromatin micro-environments for the transcription profiles they produce, we first designated cardiac- and liver-specific genes as those having 5× higher expression in the organ of interest when compared to all other tissues in the Human Protein Atlas (26). We then examined the chromatin interactions, detected in cardiomyocyte or hepatocyte Hi-C experiments, around these organ specific genes.

**Figure 1:**
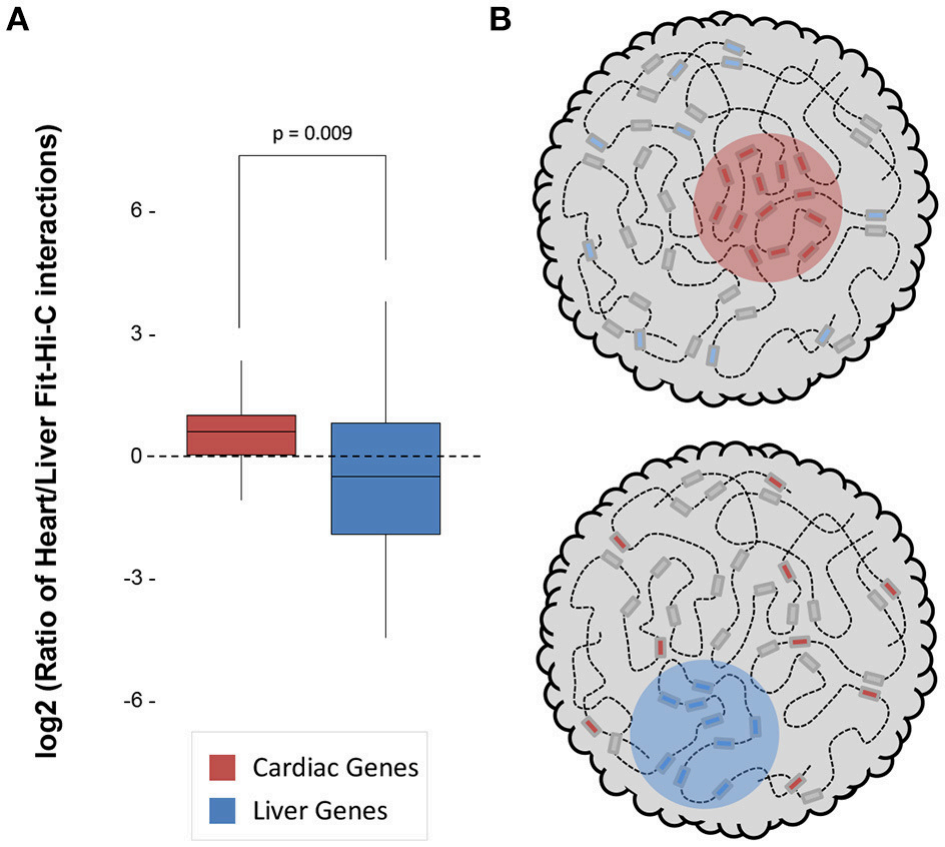
Significant Fit-Hi-C interactions are observed in organ-specific genes. **(A)** The log2 ratio of significant (*q* < 0.01) Fit-Hi-C interactions (Heart/Liver) in cardiac-specific genes (red) is higher than in liver-specific genes (blue). **(B)** Schematic demonstrating the hypothesis that regions of the nucleus containing heart-specific genes (left, red circle) contain more significant Fit-Hi-C interactions in cardiac Hi-C data when compared to liver Hi-C data (right panel and blue circle show same principle for liver genes in the liver nucleus).

Both datasets were sequenced to a similar depth (~1.3–1.5 billion read pairs; Supplementary Table 1) and achieved a similar number of significant (*q* < 0.01) intrachromosomal Fit-Hi-C interactions (115,843 in heart and 90,587 in liver; Supplementary Table 1). We quantified the log_2_ ratio of cardiac/liver Fit-Hi-C intrachromosomal interactions at cardiac or liver gene loci and found that in both organs, there was a greater ratio of interactions around that organ's specific genes (Figure 1A; *p* = 0.009). These findings suggest that structural organization in 3D underpins cell type specific transcriptomes through greater frequency of interactions (Figure 1B).

### Organ-Specific Compartmentalization Governs Heart and Liver mRNA Expression

**Figure 2:**
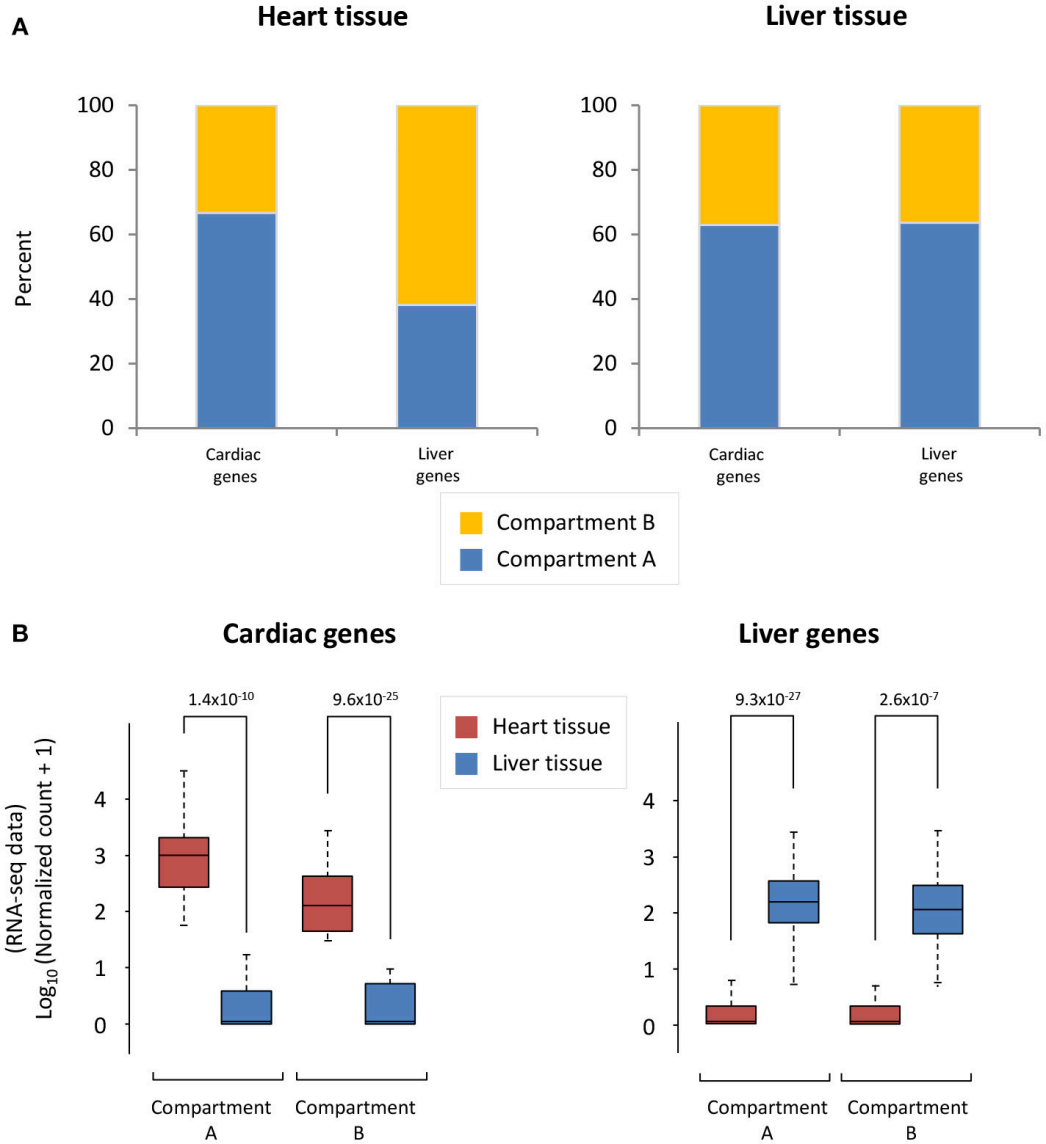
Cardiac and liver genes are situated in open compartments. **(A)** Left, in the cardiac Hi-C data, the majority of cardiac-specific genes are found in compartment A (more accessible), whereas the majority of liver genes are in compartment B (less accessible). Right, the majority of liver genes are found in compartment A in the liver Hi-C data; this is also the case for cardiac genes in liver Hi-C data. **(B)** Left, Cardiac-specific genes are more highly expressed in heart than liver genes. Right, liver-specific genes are more highly expressed in liver tissue than in the heart. The y-axis shows log_10_ of the normalized RNA-seq read counts, which are calculated according to the DESeq2 read count normalization method for each gene (see Methods). Indicated *p*-values were calculated using Wilcoxon rank-sum test. Note an observed trend of higher expression for those genes that lie within compartment A when compared to those in compartment B.

To understand the accessibility of cardiac- and liver-specific genes within the context of heart and liver chromatin, we calculated the A/B compartmentalization status of these genes as determined from Hi-C experiments. In the cardiac Hi-C data, the majority (66.7%) of cardiac-specific genes are found in compartment A (the accessible compartment), while the majority of liver-specific genes (61.9%) are found in compartment B (the less accessible compartment) (Figure 2A, left). Contrastingly, the majority of *both* cardiac- and liver-specific genes are found in compartment A in the liver Hi-C data (Figure 2A, right; 63.0% of cardiac and 63.6% of liver genes). As a positive control for the gene selections strategy, cardiac- and liver-specific genes are more highly expressed at the mRNA level in heart and liver cells, respectively (Figure 2B) in the experiments used for this study. Cardiac-specific genes in compartment A are more highly expressed than those in compartment B (*p* = 1.4 × 10^−10^ between heart and liver for genes in compartment A, *p* = 9.6 × 10^−25^ for genes in compartment B), and the same is true for liver-specific genes in liver tissue (*p* = 9.3 × 10^−27^ between liver and heart genes in compartment A, *p* = 2.6 × 10^−7^ for genes in compartment B; Figure 2B). Taken together, these data suggest that the heart contains cardiac-specific chromatin conformations that allow for cardiac (and not liver) gene accessibility and expression via a more open compartmentalization regime at specific cardiac gene loci. In contrast, liver chromatin can tolerate more cardiac specific genes in active compartments, whereas the reverse is not true for liver genes in cardiac chromatin.

### Interaction Profiles and Compartmentalization of Genes in 3D

**Figure 3:**
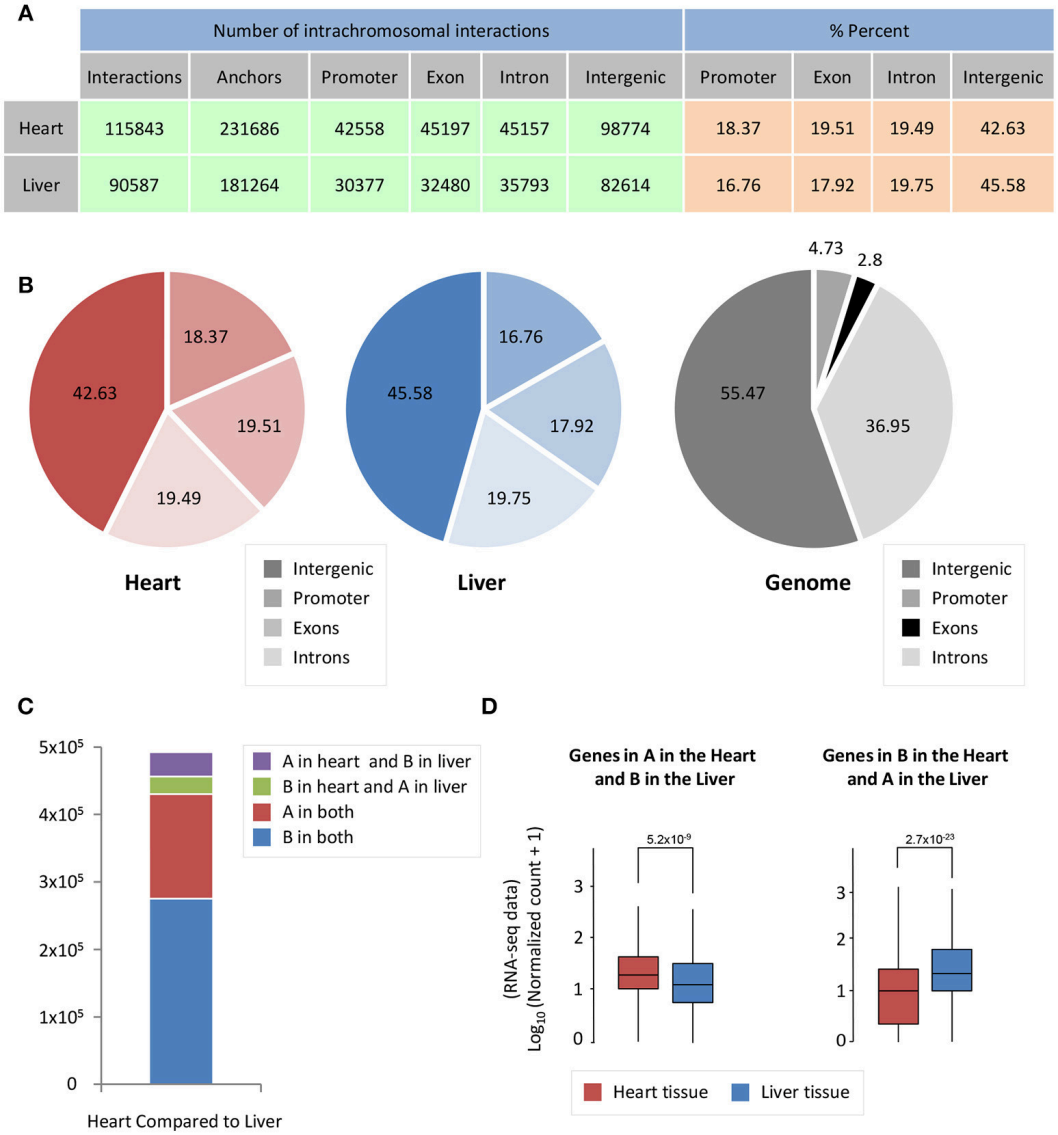
Significant Fit-Hi-C interactions have a similar distribution among annotated features of the genome. **(A)** Table describing promoter/exon/intron/intergenic distribution of Fit-Hi-C interaction anchors. The number of anchors is double the number of significant (*q* < 0.01) Fit-Hi-C interactions identified in the study. **(B)** Pie charts depicting the data shown in **(A)**. **(C)** Compartmentalization differences between heart and liver reveal that ~5% of the genome is in a different compartment between heart and liver. **(D)** The genes in compartment A in the heart and B in the liver tend to have higher expression in the heart (left, red box) than in the liver (left, blue box); in contrast, genes in compartment B in the heart and A in the liver are more highly expressed in the liver. The y-axis shows log_10_ of the normalized RNA-seq read counts, which are calculated according to the DESeq2 read count normalization method for each gene. Indicated *p*-values are calculated using Wilcoxon rank-sum test.

Cardiac- and liver-specific genes have increased accessibility and a larger number of intrachromosomal interactions at organ-specific genes. However, the distribution of intrachromosomal interactions across genomic features, as well as the compartment change of 5 kb bins in the genome, could contribute to this phenomenon in both organs. To investigate whether intrachromosomal interactions in heart and liver have different localization across genomic features (promoters, exons, introns, intergenic regions), we performed an overlap of significant intrachromosomal Fit-Hi-C interaction anchors (i.e., one side of an interaction pair) with these regions. Notably, in both heart and liver, we observe an almost identical distribution of Fit-Hi-C anchors (Figures 3A,B). Intrachromosomal Fit-Hi-C interactions are enriched within promoters and exons, and depleted from introns and intergenic regions (Figure 3B), suggesting a common packaging logic characterized by increased interactions in regions that contain genes.

To understand how A/B compartmentalization differs between heart and liver, we determined which 5 kb bins of the genome have a difference in compartment status between heart and liver. Five percent of bins are in compartment B in the heart and A in the liver, while 7% of bins are in compartment A in the heart and B in the liver (Figure 3C), for a total of 12% of the genome that shows compartmentalization differences between both organs. Genes that are in compartment A in the heart but are in compartment B in the liver are more highly expressed at the mRNA level in the heart than in the liver (*p* = 5.2 × 10^−9^, Figure 3D). Contrastingly, genes that are in compartment B in the heart and A in the liver are more highly expressed in the liver than in the cardiac RNA-seq data (*p* = 2.7 × 10^−23^, Figure 3D). Taken together, these data suggest that chromatin organization directly contributes to organ-specific gene regulation at a global scale.

### Genes With Promoter-TES Interactions Are Organ-Specific

**Figure 4:**
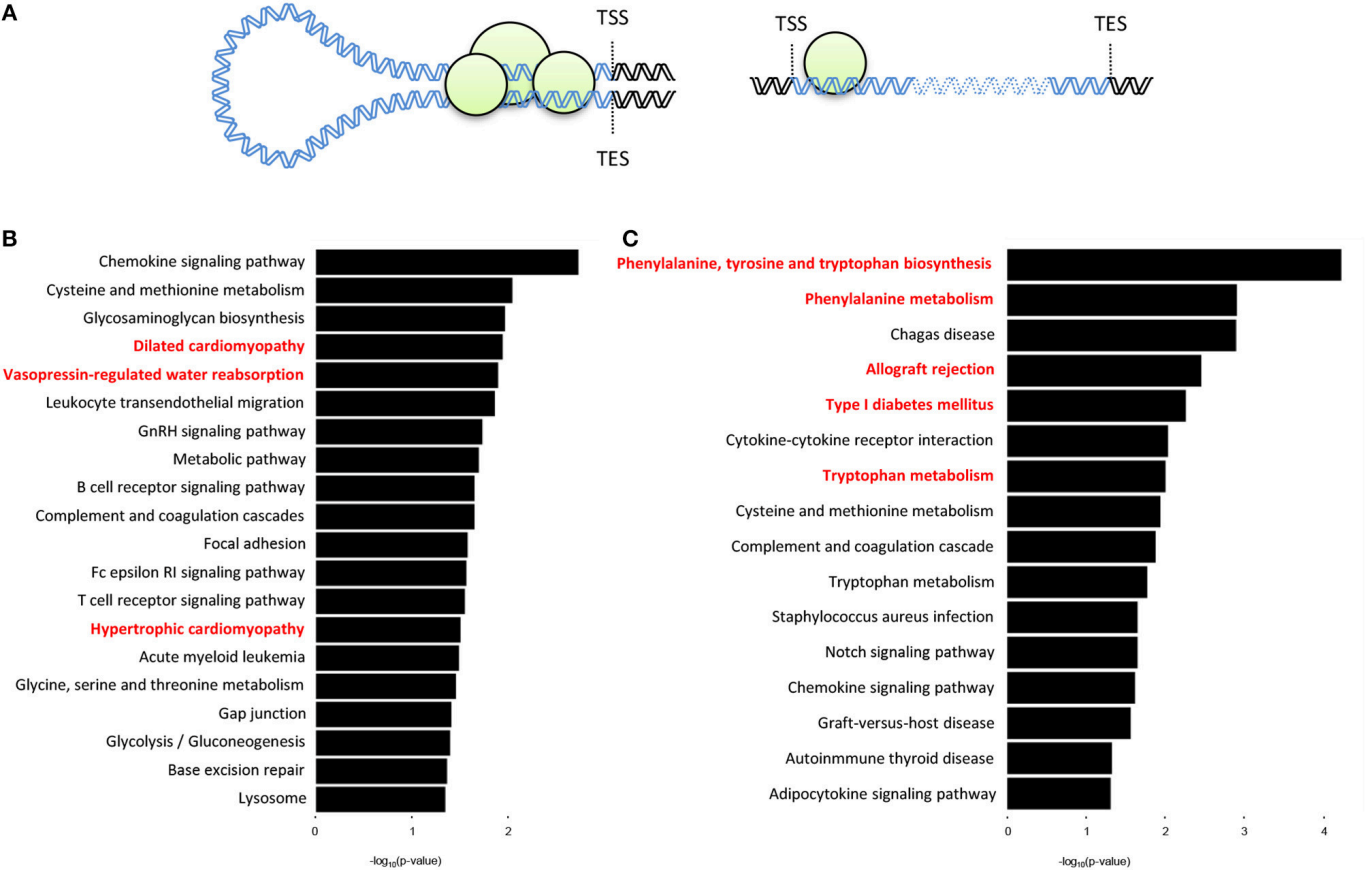
Genes with promoter-TES loops tend to have organ-specific functions within the cell. **(A)** Illustration depicting a gene with promoter-TES interactions (left) and a gene with no promoter-TES interactions (right). **(B)** KEGG pathway analysis of the genes with significant (*q* < 0.01) Fit-Hi-C interactions between promoters and transcription end sites in the cardiac Hi-C data. Cardiac-related terms are highlighted in red. **(C)** KEGG pathway analysis for genes with significant promoter-TES Fit-Hi-C interactions in liver Hi-C data. Liver-related terms are highlighted in red. For panels **(B,C)**, *p*-values are calculated using a hypergeometric test.

We next sought to determine whether there are organ-specific gene loops that govern cardiac- or liver-specific organ function. Here we define gene loops as significant (*q* < 0.01) intrachromosomal Fit-Hi-C interactions that overlap both the promoter region (−2,000 to +200 bp from transcription start site) and the transcription end site (TES) of a gene (Figure 4A). Our analyses revealed 492 and 298 genes (overlap = 78) with promoter-TES looping in the cardiac and liver Fit-Hi-C data, respectively (Supplementary Tables 2,3; note this analysis was unbiased—genes were not preselected for organ specific functions as in preceding analyses). KEGG pathway analysis on cardiac loop genes reveals enrichments for terms, such as dilated cardiomyopathy, vasopressin-regulated water reabsorption, and hypertrophic cardiomyopathy (Figure 4B) (the genes within these terms include: *Adcy6, Aqp3, Creb3l4, Des, Itgb5, Itga9, Myl2, Myl3*, and *Stx4a*). The same analysis on liver gene loops reveals enrichments for phenylalanine, tyrosine and tryptophan biosynthesis, phenylalanine metabolism, allograft rejection, and tryptophan metabolism (Figure 4C) (the genes of which include: *Cyp1a1, Cyp1a2, Fasl, Got1, H2-T10, Il2, Il12a, Lao1*, and *Tat*).

### Interchromosomal Interactions Have Different Compartment Status in Heart and Liver

**Figure 5:**
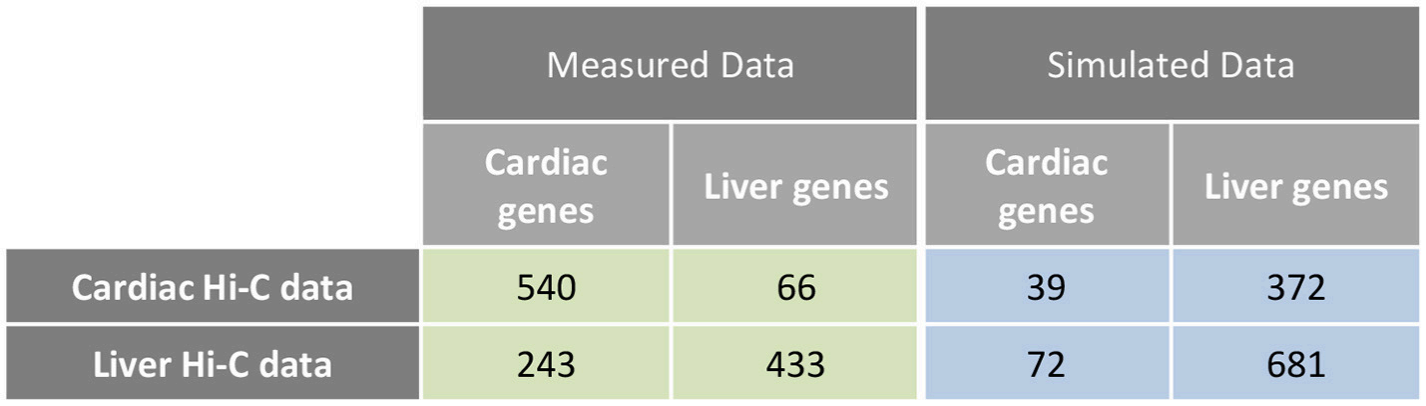
Interchromosomal Fit-Hi-C interactions are preferentially found at organ-specific genes. Analysis of interchromosomal Fit-Hi-C interactions (*q* < 0.01) reveals that more interactions are found at cardiac-specific genes in the cardiac Hi-C data, while more interactions are found at liver genes in the liver data (Measured Data, green, *p* = 2.2 × 10^−16^, Fisher's exact test). To calculate the frequency of interactions at random genes, simulations were performed on the cardiac and liver Hi-C data at random genes. Simulations were repeated 10,000 times for each cell in the blue table, and the median number of interactions at random genes was kept for statistical testing (Simulated Data, blue, *p* = 1, Fisher's exact test).

**Figure 6:**
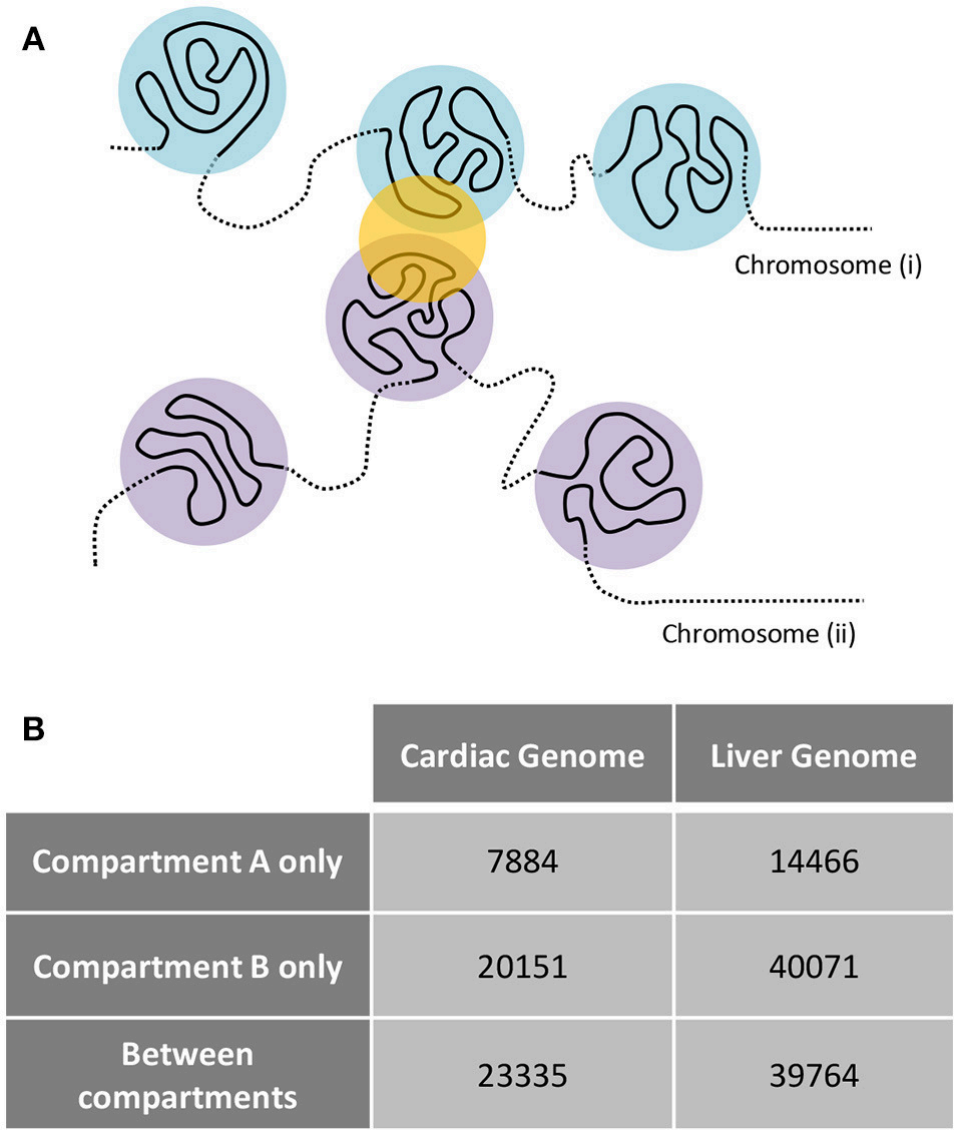
Interchromosomal Fit-Hi-C interactions bridge regions of differing A/B compartmentalization. **(A)** Concept figure showing interaction (yellow circle) between regions from different chromosomes. The regions on one chromosome (i) are shown in light blue, while regions on the other chromosome (ii) are shown in violet. **(B)** Analysis of all significant Fit-Hi-C interactions (*q* < 0.01) reveals that approximately half of interchromosomal interactions in both heart and liver nuclei act as a bridge between regions of differing A/B compartmentalization. Fit-Hi-C interactions can have both anchors interacting with compartment A or compartment B, or they can have one anchor interacting with each compartment.

Examination of interchromosomal interactions allows for exploration of regional apposition—and potentially regulation—between distinct chromosomes. Significant (*q* < 0.01) interchromosomal interactions were identified in the cardiac and liver Hi-C datasets and are summarized in Supplementary Table 1. To determine whether interchromosomal interactions from the cardiac Hi-C data preferentially overlap cardiac specific genes, we overlapped these regions with the cardiac- and liver-specific genes from the analyses in Figures 1–3. In cardiac chromatin, 540 interchromosomal interactions overlap with cardiac-specific genes, while only 63 interactions overlap with liver-specific genes. In the liver chromatin, 433 interchromosomal interactions overlap with liver-specific genes, whereas only 243 overlap with cardiac-specific genes. These data suggest that interchromosomal interactions at organ-specific genes depend on the nuclear environment within the organ of interest (*p* = 2.2 × 10^−16^, Fisher's exact test; Figure 5). To confirm this observation, we performed a simulation which resulted in no relationship between randomly selected genes from the genome and interchromosomal Fit-Hi-C interactions in either organ (*p* = 1, Fisher's exact test; Figure 5). To investigate the compartmentalization of interchromosomal interactions in cardiac and liver nuclei, we determined the compartment status at each anchor of these interactions (Figure 6A). In the cardiac Hi-C data, 7,884 significant interchromosomal interactions have both ends in compartment A, while 20,151 have both ends in compartment B, and 23,335 have each end in a different compartment (Figure 6B). In the liver Hi-C data, 14,466 significant interchromosomal interactions have both ends in compartment A, while 40,071 have both ends in compartment B, and 39,764 have each end in a different compartment (Figure 6B). In heart and liver Hi-C data, 45% and 42% of significant interchromosomal Fit-Hi-C interactions, respectively, have one end in compartment A and the other in compartment B. Stated another way, about half of significant interchromosomal interactions extend to other compartments, while the other half share compartment status. This observation suggests the existence of chromatin regions that localize to the same area in the nucleus and yet exhibit distinct compartmentalization and potentially distinct accessibility features.

**Figure 7:**
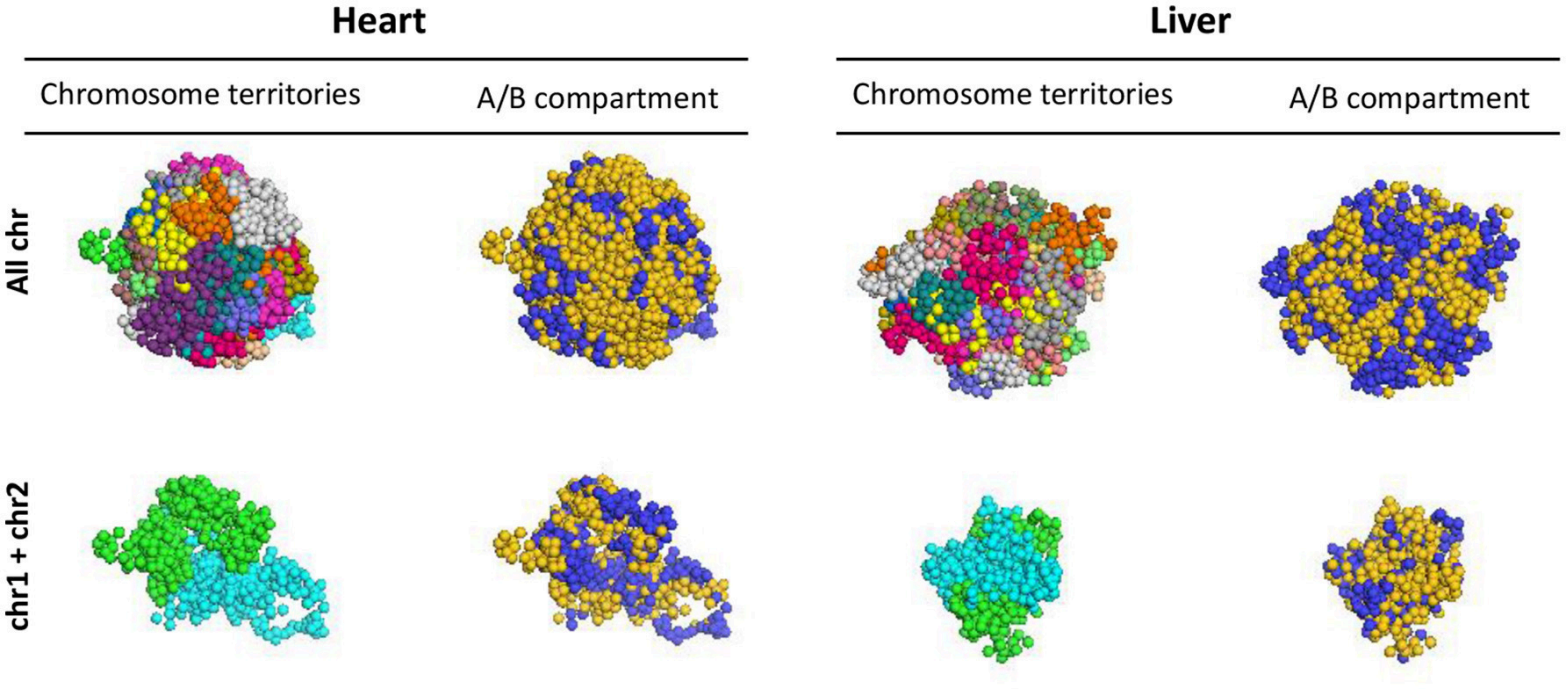
3D models of liver and heart genomes. In both heart and liver nuclei, computational models generated using PGS reveal organization of topologically associating domains (TADs) within chromosomes in 3D space (top, All chr), as well as interactions between TADs of individual chromosomes (bottom, chr1 and chr2 shown as examples). Between chromosomes, regions of concordant A/B compartmentalization can aggregate in 3D space (blue = compartment A, gold = compartment B; see Supplemental Movie 1 for 360° view of this 3D reconstruction).

HiC data is informative to define regions of local interaction, but how these substructures of the epigenome arrange in 3D has remained an open question. We performed 3D reconstruction of cardiac and liver epigenomes based on HiC data, establishing models for how chromosomes fold and for how they associate with other chromosomes, using PGS (19). The approach generates a large population of 3D genome structures, in which TAD domains are represented by spheres and are then packed into the nucleus in such a way that the formation of contacts between TAD domains is statistically consistent with the contact probability matrix from Hi-C experiments (Supplementary Figure 2). These models reveal distinct chromosomal structures within liver or cardiac epigenomes (i.e., allowing comparison of one chromosome to another), enable comparison of the individual chromosomes between organs, elucidate the surfaces of interaction between chromosomes (Figure 7; Supplementary Movie 1) and reveal insights into the spatial organization of chromatin compartments.

**Figure 8:**
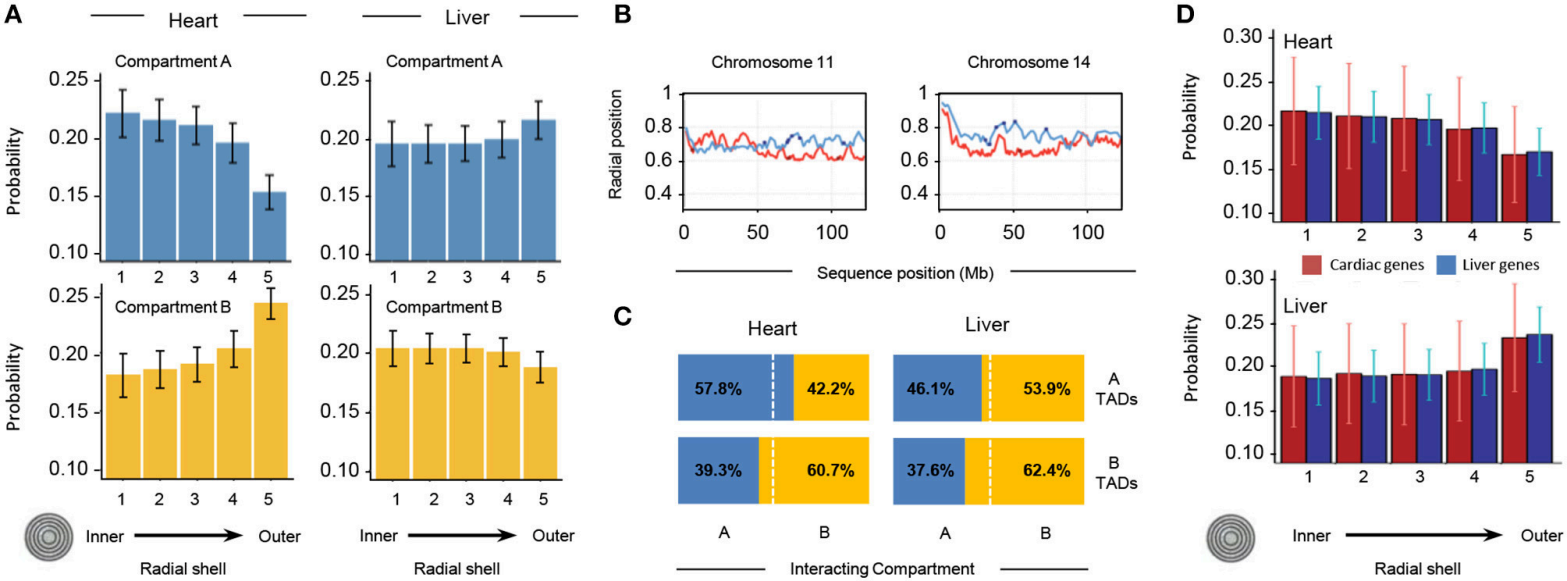
Properties of 3D models of heart and liver genomes. **(A)** The nucleus is divided into 5 concentric shells, with shell 1 in the interior and shell 5 at the periphery. In heart nuclei (left), TADs in compartment A are more likely to be found toward the interior of the nucleus and TADs in compartment B toward the periphery. In contrast, the liver Hi-C data (right) show that TADs in compartment A have a higher probability of being found toward the periphery and those in compartment B within one of the inner shells. Error bars indicate standard deviation of observations for 10,000 structures. **(B)** Across distinct regions of chr11 (left) and chr14 (right), radial positions of TADs between organs (heart in red, liver in blue) differ. Positions of heart- (red dots) and liver-specific genes (blue dots) superimposed. The y-axis shows average position (0 is the center of the nucleus, 1 indicates nuclear periphery), while the x-axis shows chromosome position in megabases. **(C)** In 3D cardiac nuclear models (left), on average 57.8% of queried A-compartment TADs form interchromosomal interactions (within 500 nm) with regions in compartment A, while 60.7% of queried B-compartment TADs form interchromosomal interactions with regions in compartment B. Contrastingly, liver models show that 53.9% of queried A-compartment TADs are within 500 nm of regions in compartment B, while 62.4% of queried B-compartment TADs interact with the same compartment in a different chromosome. **(D)** Similar analysis as in **(A)**, but with TADs that have heart- (red bars) or liver-specific (blue bars) genes. In cardiac nuclei (left), both heart- and liver-specific genes tend to associate with the nuclear center, while in liver nuclei the trend is the opposite. Error bars indicate standard deviation of observations for 10,000 structures.

To investigate the distribution of different chromatin features within the nuclear space, we divided the nuclear volume into 5 concentric shells in such a way that each shell contains 20% of the total number of TADs per structure. Based on their radial positions, all TADs in each of the 10,000 genome structures are then partitioned into the 5 shells. We then measured the probability for a TAD in a given subcompartment (A/B) to be localized in each of the concentric shells (Figure 8A). We observe striking differences in the internal organization of the compartments. In heart cells, chromatin in compartment A shows the highest localization probability in the most inner shells (shell 1 in Figure 8A), and the probability gradually decreases toward the outer regions (shell 5 in Figure 8A, top left panel). This observation is consistent with previous observations that showed highly transcribed genes to be localized toward the interior regions of the nucleus (28). Compartment B shows the opposite behavior, with the highest localization probability for the outer most shell (Figure 8A, lower left panel), consistent with the location of heterochromatin and lamina associated domains at the nuclear envelope (29, 30). In contrast, liver cells show a different spatial organization in the models. Compartment A is more evenly distributed with the highest localization probability at the outermost shell, while compartment B shows a slight decrease in localization probability toward the most outer shell.

To gain a quantitative understanding of interchromosomal TAD-TAD colocalization, we studied the compartment composition at the interchromosomal boundaries. At each TAD position, we determined all TADs that are localized within a distance of 500 nm and are part of a different chromosome. We then determined the percentage of A/B compartment found in this group of inter-chromosomal TAD neighbors. The heart genome shows a high preference for TADs in the same chromatin compartment across chromosome boundaries, indicating a high level of compartmentalization across chromosome borders. In liver cells, we observe a different behavior. While TADs in subcompartment B also show a high preference to be in proximity to TADs in same state, TADs of state A do not show a preference for the same state, showcasing the different global organization of the genome in liver nuclei.

We also calculated the average radial position of each TAD with respect to the nuclear center (Figure 8B; see Supplementary Figure 1 for comparison of all chromosomes). When plotting the average radial positions for each TAD across a chromosome we observe distinct regional differences with well-defined local minima and maxima (Figure 8B). TADs corresponding to minima are on average more interior located than directly neighboring regions in the same chromosome. These radial position profiles are markedly different for the same chromosomes in the two tissues. The correlation between the radial positon profiles is very low, and in some regions even anti-correlated (Figure 8B). These distinctions are further illustrated when examining the likelihood of regions from the same compartment to interact with each other (Figure 8C).

Finally, we examined the localization of chromatin from a gene centric view, determining the relative positioning of heart and liver specific genes in the different nuclei (Figure 8D). In agreement with the observations from Figure 8A, this gene centric analysis revealed a preference of interior localization of genes in cardiac nuclei and the antithetical behavior in liver nuclei. In summary, our structure-based calculations support the notion that, on a TAD scale (hundreds of kilobases), there are major structural differences in the global structural organization of liver and heart genomes.

## Discussion

How chromatin structure underpins gene expression has ramifications across biology and medicine. In the cardiovascular realm, as in other areas of epigenomics research, this question has largely been answered from the perspective of histone modifications (31, 32), enhancers (33), chromatin remodeling enzymes (34), transcription factors (35), DNA methylation (36), and more recently, long non-coding RNAs (37). Lacking from all of these studies has been a direct measurement of chromatin structure, rather than relying on implications of structure and accessibility as a result of the actions of other proteins or modifications. Recent chromatin conformation capture experiments (14, 38) in human and mouse cardiomyocytes now make possible examination of cardiac chromatin structure and investigation of how this structure contributes to lineage specification and heart disease.

The current study demonstrates that organ-specific genes preferentially localize in 3D in the nuclei of the organs in which they are transcribed. This conceptually straightforward hypothesis has never, to our knowledge, been tested experimentally and reveals a structural underpinning for cell type-specific transcriptomes. These observations also support the concept of transcriptional neighborhoods (39), or transcription factories, which have been hypothesized to coordinate RNA production from a select subset of DNA templates but which has never been tested in cardiovascular cell types. A caveat arising from the data used for this study (cardiac HiC and RNA-seq data were from isolated adult mouse cardiac myocytes; liver HiC data were from isolated hepatocytes and RNA-seq data from whole tissue) is that some of the cell type-specific differences in hepatocyte gene expression may be obfuscated by other cells present in the entire liver, although this should have no bearing on the analyses of chromatin architecture, which in each case were performed on an isolated cell population from adult C57BL/6J mice. Because the primary data used for these analyses were collected in two different laboratories, there is a concern that the differences in genomic organization may be attributable to confounding variables unrelated to the cell type differences. Mitigating this concern is the fact that the animals were the same genetic strain, housed in similar environments and sacrificed at the same time of day. Moreover, the sequencing data enabled identification of a comparable number of total interaction pairs in cardiac (807,707,536) and liver (701,407,381) experiments, producing interactions maps at comparable resolution (~5 kb).

Our comparison of liver and cardiac chromatin structure reveals widespread differences in compartmentalization, some but not all of which coordinate with transcriptional behaviors that vary between the organs. This finding is intriguing, given the fact that altered compartmentalization following the development of pressure overload-induced cardiac hypertrophy and failure is very minor (14): localization of genes within organ specific chromatin scaffolds is specific to cell type and resilient against pathophysiological stress. It is tempting to speculate that the differences in chromatin architecture may reflect the more proliferative nature of the liver compared to the heart. Hepatocytes, like cardiomyocytes, are terminally differentiated, and the majority of these cells—in a healthy, unstressed liver—would not be actively undergoing mitosis (and the associated genomic rearrangements). However, the liver has a well-established ability to regenerate upon physical damage and/or cell death. Perhaps the liver prepares for such an eventuality by allowing a greater number of genes to exist in accessible regions of chromatin, although further experiments will be necessary to provide evidence for this conjecture, including examination of chromatin architecture in proliferative liver tissue.

The results of the analysis of gene looping data were particularly revealing: heart and liver establish comparable numbers of promoter to TES gene loops, however this specific class of loops appears in different genes in the different organs. These findings support that at multiple scales, including the level of gene looping in addition to compartmentalization as mentioned above, structural organization of the epigenome is cell type specific.

The majority of chromatin conformation capture studies that have emerged the past few years have focused exclusively on intrachromosomal interactions. The adult cardiac myocyte, which does not divide, is an interesting test case to explore the role of interchromosomal contact surfaces in genome function—principally, although not exclusively, via gene regulation. A liver HiC dataset of comparable sequencing depth afforded the opportunity to explore contrasting features of such interactions, should they exist, within the same genome housed in separate cells' nuclei. Both epigenomes exhibited similar levels of interchromosomal interactions and in both cases, they were enriched in genes associated with the function of that cell type. Combining these interactions with 3D renderings of genomes in heart and liver provided a unique opportunity to investigate differences in chromosome folding and nuclear organization. Several observations emerged: liver and heart cells not only package their genomes differently, but they appear to obey distinct general principles of organization, wherein heart genomes preferentially localize compartment A regions toward the center and compartment B regions toward the periphery, whereas liver cells do not exhibit this behavior. Future studies will investigate whether interchromosomal interaction surfaces participate in such behaviors as cell proliferation, whether they change with age or are dependent on developmental state, and what non-DNA molecules inhabit the surfaces of interchromosomal apposition, presumably orchestrating the reproducible formation of these structures.

## Author Contributions

DC and TV conceived the study. DC performed bioinformatics and statistical analyses. DC and MR-G generated figures and diagrams. NH and FA generated and analyzed 3D genomic models. DC and TV wrote the paper. All authors approved the content of the manuscript.

### Conflict of Interest Statement

The authors declare that the research was conducted in the absence of any commercial or financial relationships that could be construed as a potential conflict of interest.

## Supplementary Material

Supplementary Table 1 Summary of Hi-C data from heart and liver.

Supplementary Table 2 List of genes with significant (*q* < 0.01) promoter-TES Fit-Hi-C interactions in the heart.

Supplementary Table 3 List of genes with significant (*q* < 0.01) promoter-TES Fit-Hi-C interactions in the liver.

Supplemental Figure 1 Radial positions of TADs differ between heart and liver. Across each chromosome, average radial positions of TADs are shown as solid lines (heart in red, liver in blue), with the positions of heart- (red points) and liver-specific genes (blue points) superimposed. The y-axis shows average radial position (0 is the center of the nucleus, 1 indicates nuclear periphery), while the x-axis shows the position of features along the chromosome.

Supplemental Figure 2 Comparison between contact probability heatmaps from experiment and structural models for heart (left) and liver (right). Each bin in the heatmap represents a TAD and each pixel represents the contact probability between 2 TADs. The lower triangle part shows the contact probability from experiment and the upper triangle shows the contact probability from the models. Contact patterns in the Hi-C experiment are very well-reproduced in the structure models. The color scale ranges from 0 probability to 0.2 probability and any probability higher than 0.2 are shown as 0.2.

Supplemental Movie 1 Movie of cardiac and liver genomes shows Figure 7 in 360 degrees. In both heart and liver nuclei, computational models generated using PGS reveal organization of topologically associating domains (TADs) within chromosomes in 3D space (top, All chr), as well as interactions between TADs of individual chromosomes (bottom, chr1 and chr2 shown as examples). Between chromosomes, regions of concordant A/B compartmentalization can aggregate in 3D space (blue = compartment A, gold = compartment B). Heart models occupy the left half of the figure, while liver models are on the right, as in Figure 7.
