# Supplementary material for: Spatial Principles of Chromatin Architecture Associated With Organ-Specific Gene Regulation
Source: Front Cardiovasc Med. 2019 Jan 15;5:186. doi: 10.3389/fcvm.2018.00186 (PMC6341059; doi:10.3389/fcvm.2018.00186)
Supplement: Supplementary Table 1 — Summary of Hi-C data from heart and liver. [file Table_1.pdf]

	Liver	Heart
Total_pairs_processed	1514557906	1362691412
Unmapped_pairs	21566557	11599265
Low_qual_pairs	0	0
Unique_paired_alignments	926874402	849101181
Multiple_pairs_alignments	408751544	385904274
Pairs_with_singleton	157365403	116086692
Low_qual_singleton	0	0
Unique_singleton_alignments	0	0
Multiple_singleton_alignments	0	0
Reported_pairs	926874402	849101181

	Liver	Heart
Valid_interaction_pairs	701407381	807707536
Valid_interaction_pairs_FF	167026398	200806229
Valid_interaction_pairs_RR	167121126	200745187
Valid_interaction_pairs_RF	167062459	199501877
Valid_interaction_pairs_FR	200197398	206654243
Dangling_end_pairs	140829780	20685150
Religation_pairs	80093214	17757504
Self_Cycle_pairs	2047729	967879
Single-end_pairs	0	0
Dumped_pairs	2496298	1983112

	Liver	Heart
Valid_interaction	701407381	807707536
Valid_interaction_rmdup	613559460	720072695
Trans_interaction	272280195	323763107
Cis_interaction	341279265	396309588
Cis_shortRange	83657373	74217618
Cis_longRange	257621892	322091970

	Liver	Heart
Fit-Hi-C interactions (q<0.01) INTRA	90587	115843
Fit-Hi-C interactions (q<0.01) INTER	94301	51370
