# Supplementary material for: Spatial Principles of Chromatin Architecture Associated With Organ-Specific Gene Regulation
Source: Front Cardiovasc Med. 2019 Jan 15;5:186. doi: 10.3389/fcvm.2018.00186 (PMC6341059; doi:10.3389/fcvm.2018.00186)
Supplement: Supplementary Table 2 — List of genes with significant (q < 0.01) promoter-TES Fit-Hi-C interactions in the heart. [file Table_2.pdf]

Genes with Promoter-TES Interaction in Cardiac Hi-C Data					
Acads	Mb	Mydgf	Evx1os	Lao1	Zdhhc22
Adam12	Mbd3	Gnl3	Med26	Oas1d	Kcng2
Adcy6	Mbl1	Tor1a	Sik3	Nsun5	Pik3c2b
Aif1	Mbl2	Tor1b	Prss41	Upk3b	4933406M09Rik
Alox12e	Meis1	Slc46a2	Zfp689	Chpf2	D430041D05Rik
Aoc3	Mertk	Lsm4	Naif1	Snx21	Kcnk15
Ap2m1	Mip	Irx4	4933433G15Rik	Ric8a	Klhdc7a
Ap4m1	Meis2	Myadm	4933440J02Rik	Tubg1	Gzmn
Aqp3	Mtx1	Ccdc97	Treml1	Tubg2	Amdhd2
Arvcf	Myl3	Ldlrad4	Rnf215	Itga9	Ssh3
Bmp10	Myl2	Arhgef25	Tmem25	Cdc42ep2	Cd207
Bsn	Nab2	Dbndd2	Mus81	Slc39a3	Mfrp
C1qb	Ncf1	Ybx2	Pitpnc1	Brms1	Mpg
Capns1	Ndufv1	Prg3	1700003E16Rik	Mrpl10	Eme1
Cbl	Nfkb2	Rab25	Dpep3	Bre	Wdr24
Cct6a	Nfkbie	Ift20	Jsrp1	Shmt2	Dpcr1
Cd19	Nefm	Gtpbp2	Tmem79	Tbx10	Orai3
Cd37	Npdc1	Cpxm1	Ephx3	Hyal3	Ccdc153
Entpd1	Nthl1	Ppp4c	Gpc2	Slc35b1	Spib
Cd72	Nxn	Ctsf	Cdca4	Akr7a5	Fam205c
Ift81	Ogg1	Arl10	Erv3	Krt5	Pced1a
Cdx2	Olfr59	Apba3	Cyb5r1	Pigh	Gm20735
Cel	Pck1	Wdr46	Entpd8	Timp4	Ablim3
Celsr1	Pcsk4	Ppp1r1a	1810062G17Rik	Mbd6	3-Mar
Ckmt1	Pde6g	Cts6	Asb6	Clic1	Grin1os
Ccr2	Prf1	Rps6kb2	Klhl40	4930433N12Rik	Zfp629
Cnih2	Plau	Pnkp	2210016L21Rik	Cts8-ps	B230319C09Rik
Cnp	Plcb2	Slc22a17	Ccdc71	Cts3	B230206H07Rik
Col14a1	Serpinf2	Jph2	Aldh1b1	Gjc2	Mical2
Cpne6	Npy4r	Myg1	C1qtnf6	Grin3b	Trmt61a
Cryab	Mapk11	Fam129a	B3gat3	Znrf1	Lgr6
Cryba4	Klk6	Rbp7	Tymp	Socs7	C130026L21Rik
Cyp17a1	Cyth2	Slc29a1	2900057B20Rik	Rassf3	Zc3h4
Cyp1a1	Psmc3	Ctsm	Prss57	BC018473	Rnf150
Dapk2	Ptp4a3	Dpysl5	Tm7sf2	Zbtb7c	Krt78
Des	Igdcc3	Slc9a3r2	1700065J11Rik	Alg3	Morn2
Dlx4	Pxn	P3h4	Izumo1	Sirt7	Lrrc24
Ebf1	Rab20	Them5	1700067G17Rik	Eif2b1	A230065H16Rik
Eef2k	Rad9a	Nicn1	Mfsd12	Irgq	Prickle4
Efna1	Rai1	Pam16	Tm9sf1	Mrgprf	Stum
Egr3	Rara	Pnpla2	Uba7	Dcaf15	Sbk2
Ehd1	Rfxank	Tmx2	Perm1	Pgghg	Gm26705
Eif2b4	Rgl2	Plin5	2310065F04Rik	Crygn	Ccdc84
Eif4a1	S100a10	Tmem134	4931428F04Rik	Mmp21	Cdsn
Eif4ebp1	Ccl22	Cep19	4931430N09Rik	Rhot2	Thoc6
Eno3	Ccl9	Med29	4930478P22Rik	Slc43a2	C730036E19Rik
Fgf8	Nptn	Gpx7	Mamstr	Iba57	Dgkeos
Fhl4	Sh2b1	Qpctl	Nkx6-3	Spag7	D630024D03Rik
Fkbp10	Ptk6	4921536K21Rik	4930488B22Rik	BC030499	Itprip
Flt3l	Slc22a12	Ccdc127	Efcab10	Gm11545	Lyrm7os
Fosb	Slfn1	Map1lc3b	4930515B02Rik	Stxbp6	Trim80
Fut4-ps1	Sstr3	Abhd15	Dusp18	Gphb5	Ly6g6f
Slc37a4	Serpina3m	1700019N19Rik	Sirt4	Susd6	Jmjd7
Gata5	Srms	Nmral1	4930591E09Rik	Acot6	Gm13119
Gba	Aurkb	Cdca5	Slc6a21	Mfsd7c	Klhdc7b
Gcgr	Bhlhe40	Mri1	Tmem234	Rrp36	Cfap73
Gjc1	Stx4a	1110004E09Rik	Scarletltr	Apbb3	Gm15713
Gnat1	Tagln2	Lurap1	St5	Catsper1	D330041H03Rik
Rack1	Tcea2	Sdccag3	2510039O18Rik	Npas4	Gm7538
Grk5	Prdx2	Pomgnt1	Klhl2	Cfap157	Gm10433
Grb2	Tgfbr3	Crip2	9930111H07Rik	Large2	Gm16287
Hlx	Thy1	0610030E20Rik	Spata31	Trib3	D4Ertd617e
Nr4a1	Tlx1	Fam189b	Creb3l4	Hcrtr1	Gm13648
Hspa1l	Traf5	Wipf2	2210407C18Rik	Man1c1	Rpl37rt
Hsd11b2	Tuba1c	Syf2	Fam131a	Fbxo44	Gm10814
Hsp90ab1	Uck1	Fbxw9	B430010I23Rik	Plekhn1	Gm19510
Il11	Upk3a	Slc44a2	Cxcr6	Sds	Gm15408
Ilf3	Cdh23	Trp53inp2	Kat5	Fam109a	Gm13003
Ilk	Vav2	Acss1	Ankrd17	Mat2a	Cbarp
Irf1	Vegfb	Taf11	Wdr6	Arhgap35	Gm29805
Itgb5	Fmnl3	Gsdmd	Il24	Klc3	Gm12348
Kcna7	Xbp1	1700003D09Rik	Pcdhgc3	Tbc1d17	LOC105246506
Kcnab3	Ywhag	Tfpt	Arid1a	Prr14	
Kcnj12	Rnf112	2410004I01Rik	Cnnm2	Ccdc189	
Kcnk4	Zfp46	Nabp2	Tinagl1	Csgalnact1	
Krt33b	Zp3	Spaca9	Ptges2	Ces2b	
Krt1	Car14	Srrd	D930048N14Rik	Elmo3	
Krt84	Pappa2	Hk1os	Nol12	Carmil2	
Krt4	Impdh2	2010107E04Rik	Deptor	Cdk10	
Krt8	Mapk7	Plscr3	Phlpp1	6030466F02Rik	
Lat	Dazap2	Cactin	Ehd4	Atg4d	
Lhx3	B9d1	Reep6	Abtb2	Stac3	
Mycl	Naglu	Kptn	Tchh	Pla2g3	
Mafg	Nagpa	Dhrs13	Fam46b	Fscn2	
