# Supplementary material for: Spatial Principles of Chromatin Architecture Associated With Organ-Specific Gene Regulation
Source: Front Cardiovasc Med. 2019 Jan 15;5:186. doi: 10.3389/fcvm.2018.00186 (PMC6341059; doi:10.3389/fcvm.2018.00186)
Supplement: Supplementary Table 3 — List of genes with significant (q < 0.01) promoter-TES Fit-Hi-C interactions in the liver. [file Table_3.pdf]

Genes with Promoter-TES Interaction in Liver Hi-C Data			
Acr	Stat3	Efcab10	Gzmn
Aif1	Wnt8a	4930515B02Rik	Cd207
Alox12e	Sdc4	Spaca6	Slc5a2
Aqp2	Tfeb	Prr3	Olfr183
Aqp3	Tubb4a	4930555B11Rik	Olfr1414
Bsn	Ywhag	Ankrd53	Trim61
C1qb	Rnf112	Slitrk5	Krt12
C3	Zfp93	4930591E09Rik	E030025P04Rik
C4b	Pappa2	Rnf183	Mir124a-1hg
Cd24a	Fiz1	Fbxo31	Dpcr1
Cd37	Prnd	Got1l1	Zfp384
Cd82	Ing4	Slc6a21	Tm4sf19
Celsr1	Gnl3	St5	Klk15
Ccr1	Irx4	Fam162b	Klk14
Ccr5	Ech1	9930111H07Rik	A830011K09Rik
Col10a1	Lurap1l	B230112J18Rik	Ablim3
Cpne6	Prg3	Ccdc190	4732490B19Rik
Crx	Rab25	Tmem247	Tmem91
Csk	Chst2	Nol3	Il1bos
Cst7	Prss16	B430010I23Rik	Zfp78
Cyp1a1	Irgm2	Fbxo34	Serpina11
Cyp1a2	Il1f5	Dtx3	Gm1587
Des	Cts7	Siglece	Sbk3
Dhh	Cpxm1	Trem2	Sbk2
Dnase1l3	Dmrtb1	Pcdhgb8	Gm26705
Ecm1	Ick	Pcdhga12	Serpinb3d
Edn2	Kcne3	Pcdhb16	Krt87
Eno2	Asic5	Tchh	Lyrm7os
Ephx2	Ppp1r1a	Lao1	Ankrd13c
Fasl	Cts6	Oas1d	Gm1965
Fga	Rnf130	Upk3b	Tmem92
Flt3l	Gmfg	AI314278	Arsi
Fut4-ps1	Ctsm	Fam110c	Gpr17
Gjc1	Dpysl5	Gmpr2	9230009I02Rik
Gnl1	Gkn2	Mmrn2	Zfp551
Gna12	Ubxn6	Krt9	Gm11437
Gna15	Cep19	Rnf169	Angptl7
Got1	Ceacam11	R3hdm4	Gm16287
Lpcat3	Nde1	Pgc	D4Ertd617e
H2-Q4	Kcnv1	Krt5	Gm4719
H2-T10	Rnf41	Pigh	Krt83
Hpx	Polr2g	Timp4	Gm12505
Igfbp6	Sec14l2	Cd209d	Gm19345
Il12a	Nmral1	Cd209a	A730020E08Rik
Il2	Snrpg	Gpr37l1	Gm29805
Irx1	A930003A15Rik	Rassf3	Gm29669
Irx2	Rab13	BC018473	
Itih1	Tmem216	Pom121l2	
Kcna4	Nadk2	Dtx4	
Serpina3c	Lyzl6	Ghsr	
Krt19	2310034O05Rik	Zfp710	
Krt33b	Tatdn1	Best2	
Krt86	Prss32	Pth2r	
Krt84	Nabp2	Slc43a2	
Krt2	Spaca9	Slc36a1	
Mfap2	2610528A11Rik	Gm38403	
Fxyd3	Hk1os	Mgl2	
Cxcl9	2010107E04Rik	Trim25	
Myl3	Gfod2	Gm11545	
Myl2	Krt28	Mfsd7c	
Neurl1a	4933432G23Rik	Zfp503	
Nefm	Dmrtc2	Shisa2	
Nhlh2	4933430N04Rik	Rtp2	
Nov	4933433G15Rik	Speer2	
Nr4a2	Susd2	AI661453	
Nxn	Dpep3	Dpp9	
Pax4	Dnmbp	1110051M20Rik	
Pck1	Erv3	Gimap7	
Penk	Gcnt3	Lypd4	
Pomc	Spata31d1a	Acsm2	
Klk6	1700021F07Rik	Thumpd1	
Ptcra	1700030O20Rik	Ces2b	
Qk	Ces2g	Tat	
Rcvrn	Tagap	6030466F02Rik	
Resp18	Smc2os	Apeh	
Stmn3	Rassf6	Prss42	
Ccl6	Bpifa3	Pla2g3	
Sfrp2	1700067G17Rik	Spata31d1b	
Nptn	4930428G15Rik	Lrit1	
Slc31a1	Zswim5	Neu4	
Slfn1	Ifitm7	Kcng1	
Serpina3g	Mamstr	Cela3a	
Serpina3n	4930448H16Rik	Nyap1	
Spic	4930503B20Rik	Pskh1	
