# Supplementary figures and images for: Spatial Principles of Chromatin Architecture Associated With Organ-Specific Gene Regulation
Source: Front Cardiovasc Med. 2019 Jan 15;5:186. doi: 10.3389/fcvm.2018.00186 (PMC6341059; doi:10.3389/fcvm.2018.00186)

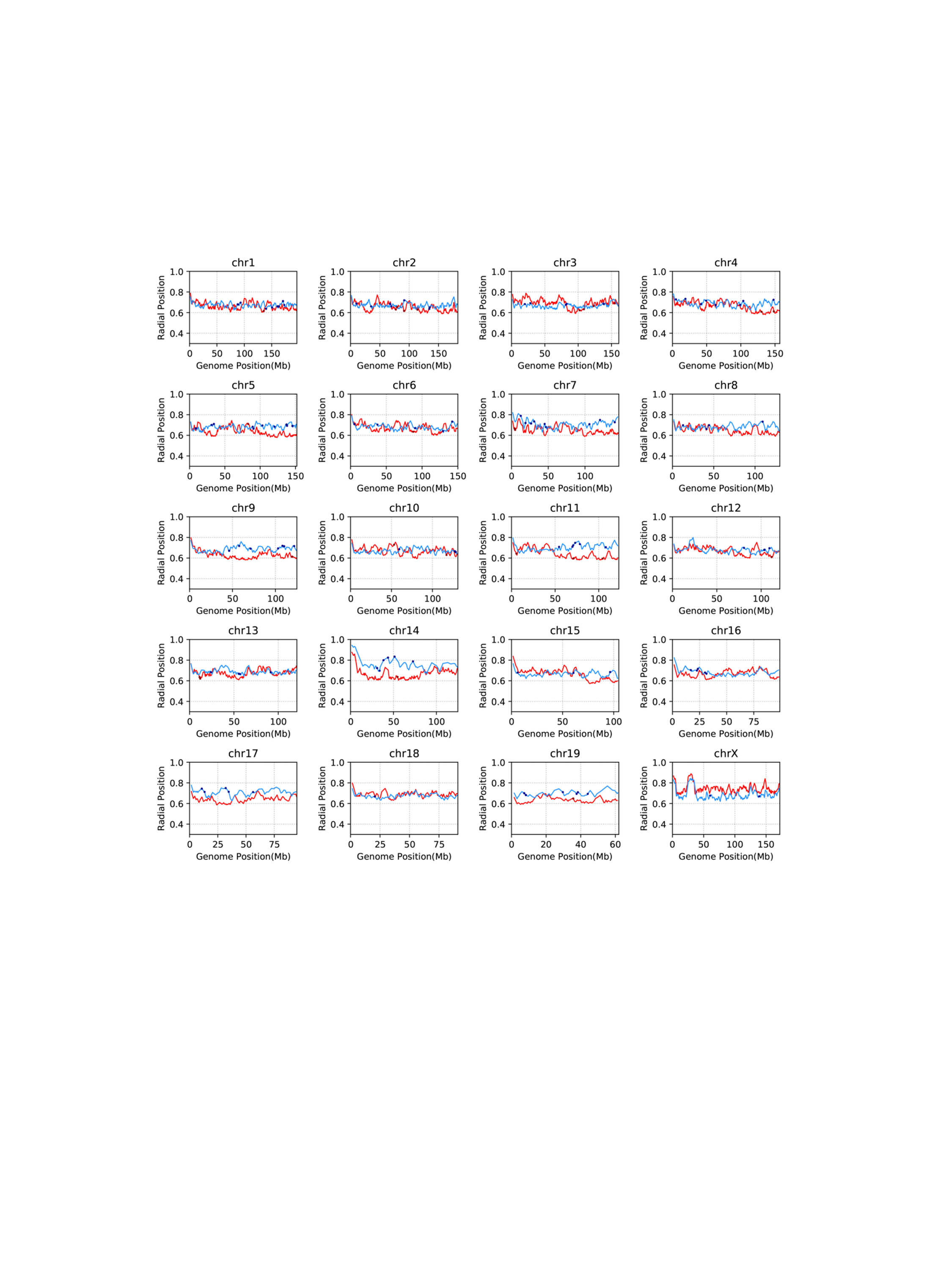

Supplement: Supplemental Figure 1 — Radial positions of TADs differ between heart and liver. Across each chromosome, average radial positions of TADs are shown as solid lines (heart in red, liver in blue), with the positions of heart- (red points) and liver-specific genes (blue points) superimposed. The y-axis shows average radial position (0 is the center of the nucleus, 1 indicates nuclear periphery), while the x-axis shows the position of features along the chromosome. [file Image_1.tif]

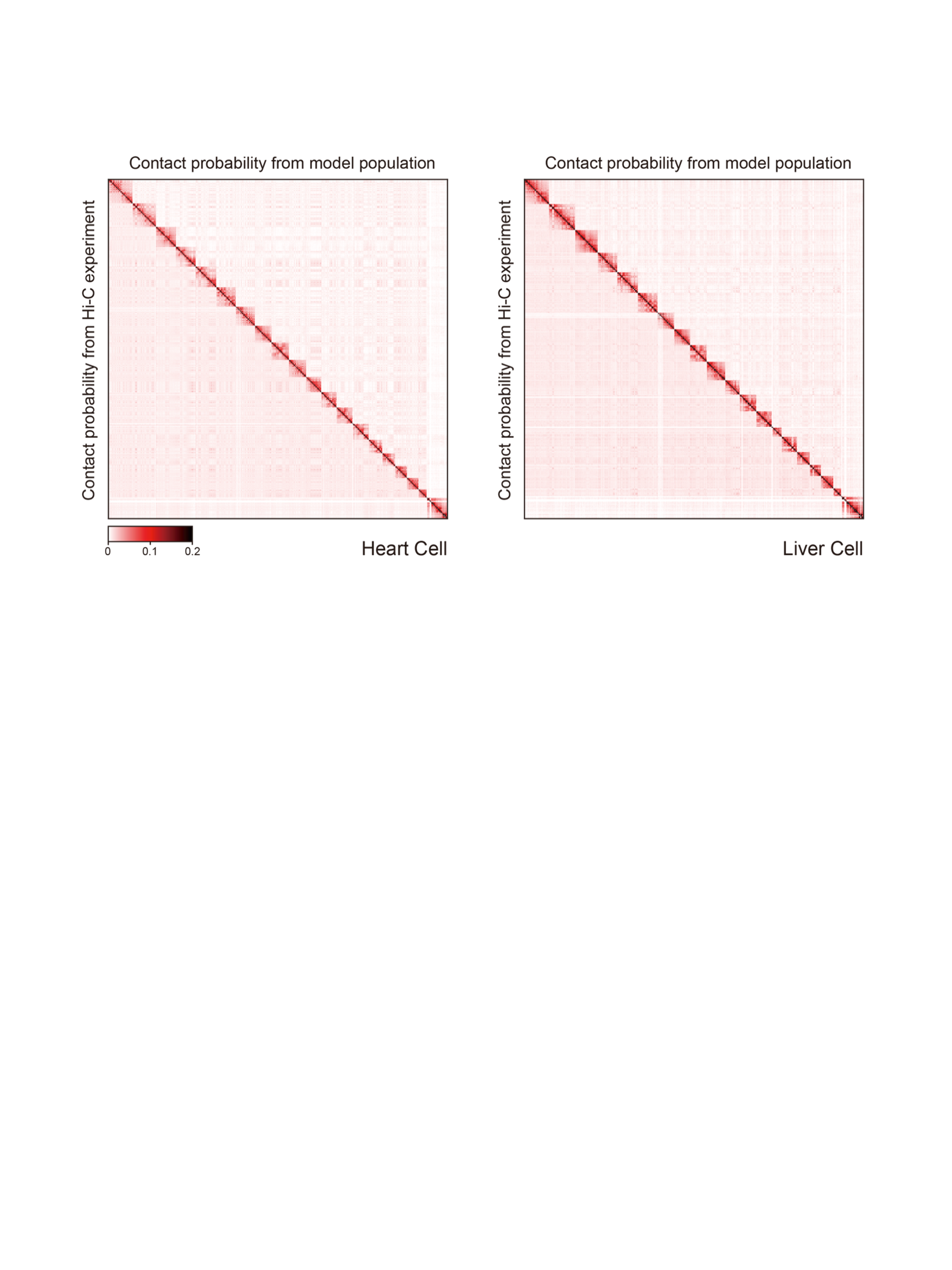

Supplement: Supplemental Figure 2 — Comparison between contact probability heatmaps from experiment and structural models for heart (left) and liver (right). Each bin in the heatmap represents a TAD and each pixel represents the contact probability between 2 TADs. The lower triangle part shows the contact probability from experiment and the upper triangle shows the contact probability from the models. Contact patterns in the Hi-C experiment are very well-reproduced in the structure models. The color scale ranges from 0 probability to 0.2 probability and any probability higher than 0.2 are shown as 0.2. [file Image_2.tif]
